# Sivelestat (Neutrophil Elastase Inhibitor) as an Anti-inflammatory and Anti-viral Agent: An In Silico Study

**DOI:** 10.7759/cureus.56846

**Published:** 2024-03-24

**Authors:** Divya Rajagopal, Radhakrishnan Narayanaswamy, Vasantha-Srinivasan Prabhakaran

**Affiliations:** 1 Pharmacology, Saveetha Medical College and Hospital, Saveetha Institute of Medical and Technical Sciences, Chennai, IND; 2 Biochemistry, Saveetha Medical College and Hospital, Saveetha Institute of Medical and Technical Sciences, Chennai, IND; 3 Bioinformatics, Saveetha School of Engineering, Saveetha Institute of Medical and Technical Sciences, Chennai, IND

**Keywords:** influenza neuraminidase, chikungunya nsp2 protease, matrix metalloproteinase, sivelestat, molecular docking

## Abstract

Background

Sivelestat is a potent and specific neutrophil elastase inhibitor. It is clinically used in treating lung injury and respiratory distress syndrome. This engaged us to undertake the present study in which sivelestat was studied as an anti-inflammatory and anti-viral agent.

Methodology

The docking study of sivelestat on matrix metalloproteinase-2 (MMP-2), matrix metalloproteinase-9 (MMP-9), chikungunya virus nonstructural protein-2 (CVnsP2) protease, and influenza A (H1N9) virus neuraminidase was assessed using the Chemistry at Harvard Macromolecular Mechanics (CHARMM) Dock (CDOCK) method. Furthermore, molecular physicochemical; bioactivity; absorption, distribution, metabolism, and excretion (ADME); toxicity; and Search Tool for Interacting Chemicals (STITCH) analyses were performed by using the Molinspiration (Molinspiration Cheminformatics, Slovensky Grob, Slovak Republic), SwissADME SwissADME (Swiss Institute of Bioinformatics, Quartier Sorge - Bâtiment Amphipôle, Switzerland), pkCSM (University of Melbourne, Melbourne, Australia), and STITCH-free online tools.

Results

The molecular physicochemical assessment of the ligand (sivelestat) showed no (zero) violation and agreed with the thumb rule of five, otherwise known as Lipinski’s rule of five. ADME prediction of the ligand (sivelestat) is shown to possess a low gastrointestinal absorption (GIA) property. Similarly, toxicity analysis of the ligand (sivelestat) is predicted to have a hepatotoxicity effect. STITCH analysis reveals that the ligand (sivelestat) has exhibited interactions with the three human proteins.

Conclusions

The present molecular docking studies showed that the ligand (sivelestat) has successfully docked with all four enzymes of interest. Hence, the current finding has provided a good understanding of sivelestat as an effective suppressor activity against all four enzymes: MMP-2, MMP-9, CVnsP2 protease, and influenza neuraminidase.

## Introduction

Inflammation is a direct tissue response to injury or pathogenic infection caused by external or internal stimuli [1]. The inflammatory stimuli have four types based on the nature of stimuli: physical stimuli such as ultraviolet radiation and X-rays, chemical stimuli such as carrageenan and croton oil, infective stimuli such as Propionibacterium acnes, and immunological stimuli namely auto-immune reaction/response such as rheumatoid arthritis [2].

Physical, chemical, and infective stimuli are caused mainly by external stimuli, whereas internal or endogenous stimuli cause immunological stimuli. Infective and immunological stimuli are otherwise called biological stimuli. Inflammation is broadly classified into two types: a) acute and b) chronic inflammation. Acute, or short-term type of inflammation, is the immediate response of a tissue to damage. Chronic, or long-term type of inflammation, is a prolonged inflammatory response at the inflammation site. In the course of inflammation, diverse types of serine proteinases are involved. For instance, mast cells release trypsin and tryptase enzymes, while neutrophils induce enzymes such as neutrophil elastase, cathepsin-G, and proteinase-3 [3]. Similarly, matrix metalloproteinases (MMPs) have been involved in almost all human diseases in which inflammation is present [4,5]. MMPs have been shown to exhibit both pro- and anti-inflammatory activities [2].

Several studies have reported the importance of MMPs in acute inflammation. If these MMPs are arrested or inhibited, progression to chronic inflammation and resultant tissue damage may be avoided [6]. Antiviral drugs are agents that inhibit the spread of the virus by preventing entry to host cells, arresting viral protein synthesis, and preventing genome replication. Interestingly, today, a few proteolytic viral enzymes (such as viral polymerase, integrase, and reverse transcriptase) serve as targets for developing new antiviral agents [7]. 

Sivelestat is a well-known neutrophil elastase inhibitor developed and manufactured by Ono Pharmaceutical Company in Osaka, Japan. It is currently used in Japan and the Republic of Korea for treating acute lung injury (ALI ) including Acute Respiratory Distress Syndrome (ARDS) in patients with Systemic Inflammatory Response Syndrome (SIRS) and its application has been investigated in several clinical trial studies [8]. Sivelestat has been shown to reduce both neutrophil elastase levels and interleukin 6 (IL-6) production [9]. Furthermore, inhibiting human neutrophil elastase (HNE) is a new approach for treating inflammatory lung diseases, such as swine flu (H1N1) and severe acute respiratory syndrome (SARS) virus infections [10].

This prompts us to perform the current investigation where sivelestat was assessed on the molecular docking behavior of matrix metalloproteinase-2 (MMP-2) and matrix metalloproteinase-9 (MMP-9), chikungunya virus nonstructural protein-2 (CVnsP2), and influenza A (H1N9) virus neuraminidase using CDocker method. Moreover, a) molecular physicochemical, b) bioactivity, c) absorption, distribution, metabolism, and excretion (ADME), d) toxicity, and e) Search Tool for Interacting Chemicals (STITCH) analyses were determined using Molinspiration (Molinspiration Cheminformatics, Slovensky Grob, Slovak Republic) (a and b); SwissADME (Swiss Institute of Bioinformatics, Quartier Sorge - Bâtiment Amphipôle, Switzerland) (c); pkCSM (University of Melbourne, Melbourne, Australia)(d) and STITCH free online tools, respectively. Hence, the current investigation aimed to assess the sivelestat as an anti-inflammatory (MMP-2 and MMP-9 inhibitor) and anti-viral (chikungunya and influenza viruses) agent using the in silico method.

## Materials and methods

Ligand preparation

The chemical structure of the ligand (sivelestat, ID 96875) was downloaded from the ChemSpider database (Royal Society of Chemistry, Cambridge, United Kingdom). The ligand (sivelestat) was drawn in ChemDraw (CambridgeSoft, Cambridge, USA) and energy minimization (EM) of the ligand was performed by using ChemDraw3D. This energy-minimized structure was further subjected to in silico studies [11].

Target enzyme identification and preparation

The 3D structures of the MMP-2 (PDB^■^ number: 1QIB with 2.80 Å resolution); MMP-9 (PDB^■^ number: 4H1Q with 1.59 Å resolution); CVnsP2 protease (PDB^■^ number: 3TRK with 2.39 Å resolution), and H1N9 virus neuraminidase (PDB^■^ number: 1L7F with 1.8 Å resolution) were obtained from ^■^Protein Data Bank database. The A chain of these enzymes was prepared individually by deleting other chains (B, C, and D), ligands, and crystallographically observed water (water without hydrogen bonds). The four target enzymes stated above were prepared by adopting the UCSF Chimera free software tool (Regents, University of California, San Francisco, USA) [7].

Molecular physicochemical and bioactivity score determination

The molinspiration free web database was applied to calculate 13 descriptors: logP, total polar surface area (TPSA), molecular weight, number of atoms (Natoms), number of O or N (NHBA), number of OH or NH (NHBD), number of rotatable bonds (Nrotb), number of violations to Lipinski’s rule (Nviolations), molecular volume, G protein-coupled receptors (GPCR) ligand, ion channel (IC) modulator, kinase inhibitor (KI), and nuclear receptor ligand (NRL) [12].

ADME properties analysis

ADME analysis was assessed by applying the SwissADME free web tool [12].

Toxicity analysis

Toxicity analysis for sivelestat (ligand) was determined using the pkCSM free web server, predicating the small molecule or ligand pharmacokinetic properties using the graph-based signature method [13].

STITCH analysis

In the present investigation, the STITCH free web server was utilized for analyzing the interaction between sivelestat (ligand) and proteins of the target organism. In this case, Homo sapiens was chosen as the target organism [11,14].

Docking studies

Molecular docking was performed on the crystal structures of four target enzymes MMP-2, MMP-9, chikungunya nsP2 protease, and influenza neuraminidase obtained from Protein Data Bank and subsequently, CDOCKER protocol under the Protein-Ligand interaction section was utilized for docking study. The CDOCKER is a grid-based molecular docking approach that utilizes Chemistry at Harvard Macromolecular Mechanics (CHARMM) force fields. Proteins/enzymes were kept rigid (R) whereas the ligands were retained to flexible (F) during the refinement. Two hundred random ligand conformations were then generated from the initial ligand structure through high-temperature molecular dynamics followed by random rotations, refinement by grid-based (GRID 1) simulated annealing, and a final grid-based or full force field minimization (FFFM).

In this study, the ligand was heated to the temperature of 700 K (in 2000 steps). The cooling steps were set to 5000 steps to 300 K (cooling temperature). The grid extension was set to 10 Å, and hydrogen (H) atoms were added to the structure and all ionisable residues were set at their regular protonation state of a neutral pH [7]. For the chosen ligand (sivelestat), the best 10 ligand binding poses were ranked according to their CDOCKER binding energies. The binding site interactions were predicted from the top among the ten ligand binding poses and further top ligand binding pose was subjected to in situ ligand minimization (ILM). Finally, the binding site interaction analysis was performed using Discovery Studio Visualizer (Dassault Systemes BIOVIA, San Diego, USA). [7,11].

## Results

In the current investigation, the sivelestat (ligand) showed nil violation of Lipinski’s rule of five (Table 1).

**Table 1:** Molecular physicochemical analysis of sivelestat using Molinspiration free web server Log A^■^: Octanol-Water partition coefficient; TPSA^◊^: Total polar surface area; Natoms^*^: Number of non-hydrogen atoms; nON^▲^: Number of hydrogen bond acceptors (O and N atoms); ׁ nOH NH: Number of hydrogen bond donors (OH and NH groups); Nviolations^◘^: Number of rule of five violations; Nrotb**: Number of rotatable bonds

Ligand	Log A^■^	TPSA^◊^	Natoms^*^	Molecular Weight	nON^▲^	nOH NH ^ׁ^	Nviolations^◘^	Nrotb^**^	Molecular Volume
Sivelestat	1.23	138.87	30	434.47	9	3	0	9	368.63

The sivelestat (ligand) showed an active bioactivity score towards two descriptors (nuclear receptor ligand and protease inhibitor). Interestingly, against other descriptors, it showed a moderate active score (Table 2).

**Table 2:** Bioactivity score analysis of sivelestat using Molinspiration free web server GPCR◊: G protein-coupled receptors; IC: Ion channel; KI: Kinase inhibitor; NRL: Nuclear receptor ligand; PI: Protease inhibitor; EI: Enzyme inhibitor

Ligand	GPCR^◊^ Ligand	IC Modulator	KI	NRL	PI	EI
Sivelestat	-0.07	-0.19	-0.37	0.04	0.30	-0.01

The ligand (sivelestat) exhibited a low gastrointestinal absorption (GIA) effect (Table 3).

**Table 3:** ADME (Pharmcokinetic) analysis of sivelestat using SwissADME free web server GlA^◊^: Gastrointestinal absorption; BBB^◘^: Blood brain barrier permeant; P-gp^▼^: P-glycoprotein substrate; CYP: Cytochrome P450 Inhibitor; ADME: Absorption, distribution, metabolism and excretion

Ligand	GlA^◊^	BBB^◘^	P-gp^▼^	CYP1A2^*^	CYP2C19^*^	CYP2C9^* ^	CYP2D6^*^	CYP3A4^*^	Log Kp^▲ ^
Sivelestat	Low	N**	N**	N**	N**	Yes	Yes	N**	-6.8

In addition to this, sivelestat predicted to inhibit two cytochrome P450 enzymes such as CYP2C9 and CYP2D6. The ligand (sivelestat) predicted to have hepatotoxicity (Table 4).

**Table 4:** Toxicity analysis of sivelestat using pkCSM free web server AT: AMES toxicity; ^◘^hERG-1: Human ether-a-go-go-related gene inhibitor-1; ^◊^hERG-2: Human ether-a-go-go-related gene inhibitor-2; ^■^SS: Skin sensitization; ORAT^▲^: Oral rat acute toxicity (lethal dose LD50 in mol/kg); ^▼^MT: Minnow toxicity (log mM)

Ligand	^ׁ^AT	^◘^hERG-1	^◊^hERG-2	Hepatotoxicity	^■^SS	ORAT^▲^ (LD_50_)	^▼^MT
Sivelestat	No	No	No	Yes	No	2.414	0.263

**Figure 1:**
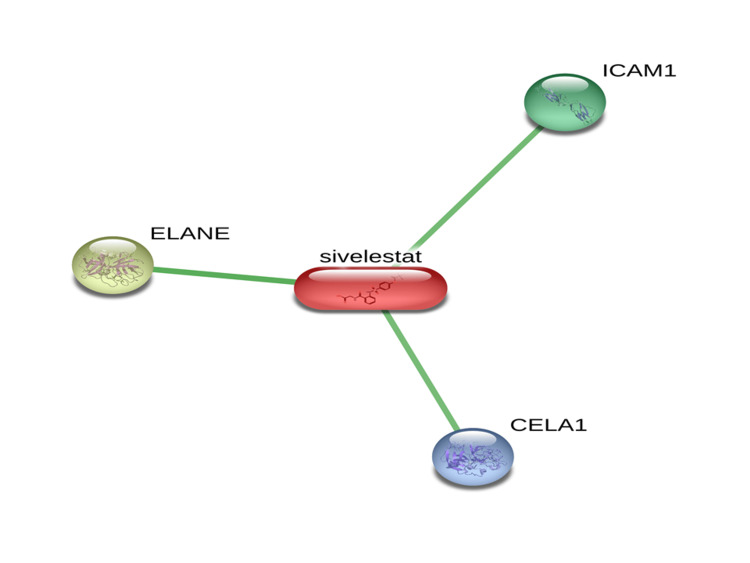
Sivelestat (ligand) interaction with the human proteins using STITCH free web server CELA 1: Chymotrypsin-like elastase; ELANE: Elastase, Neutrophil Expressed; ICAM 1: Intercellular Adhesion Molecule 1

STITCH analysis of the ligand (sivelestat) had shown interaction with the three human proteins Chymotrypsin-like elastase (CELA 1); Elastase, Neutrophil Expressed (ELANE); and Intercellular Adhesion Molecule 1 (ICAM 1) (Figure 1).

**Figure 2:**
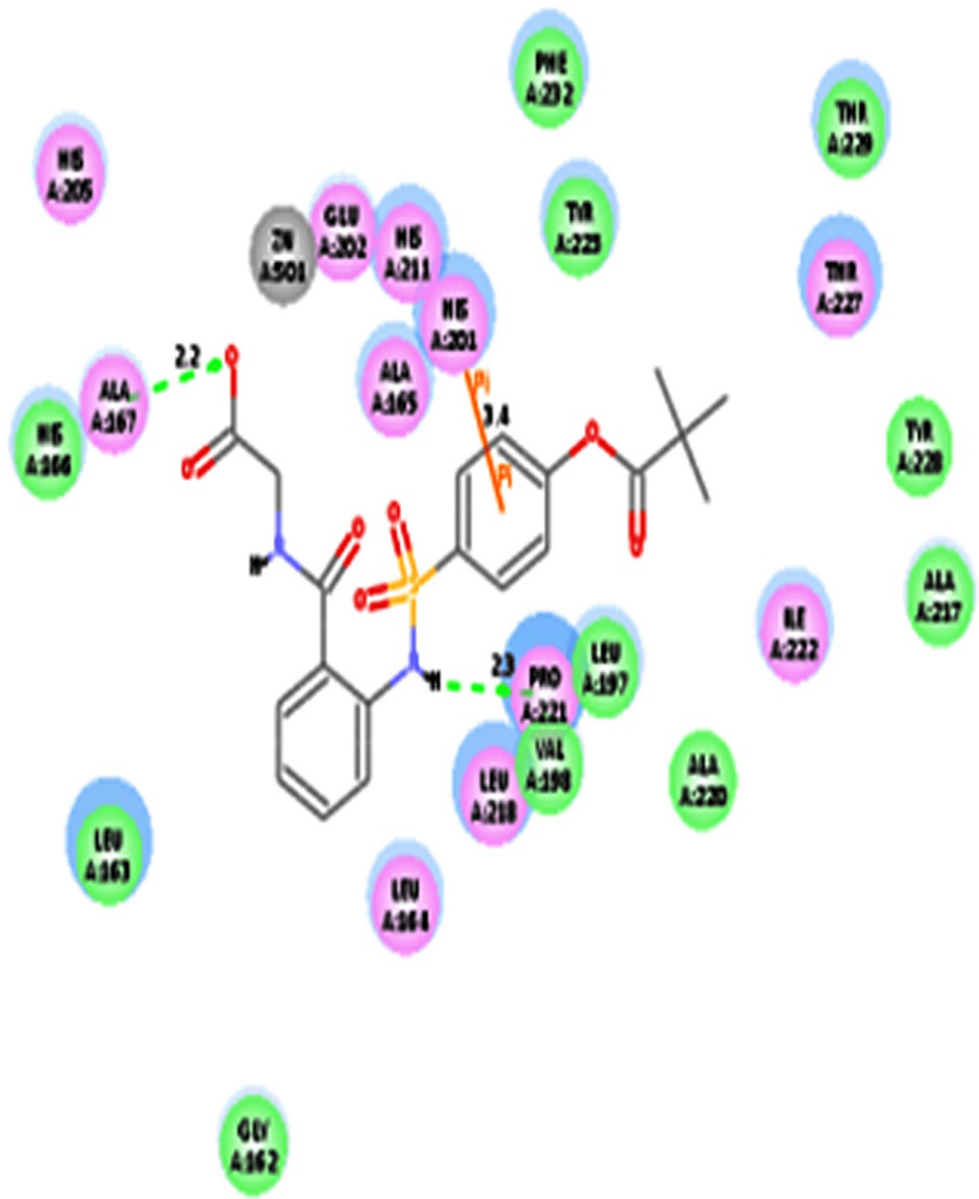
Interaction analysis of sivelestat (drug) with that of MMP-2 Hydrogen atoms have been excluded in the 2D figure to improve precision. Bond distances (BD) were indicated in angstroms (Å) units. MMP-2: Matrix metalloproteinase-2

In the present study, ligand (sivelestat) showed the interaction energy (-76.4 kcal/mol) and also showed three AA residues (Ala167, His201, and Pro221) interactions with MMP-2 (Table 5 and Figure 2).

**Table 5:** CDOCKER IE analysis of ligand (sivelestat) with MMP-2 using CDOCKER method IE: Interaction energy; AA: Amino acid MMP-2: Matrix metalloproteinase-2; ◊: Pi-Pi interaction

Ligand	CDOCKER IE (-kcal/mol)	Interaction of AA residue	Bond distance in Å
Sivelestat	76.4	Ala167	2.2
		Pro221	2.3
		His201^◊^	3.4

**Figure 3:**
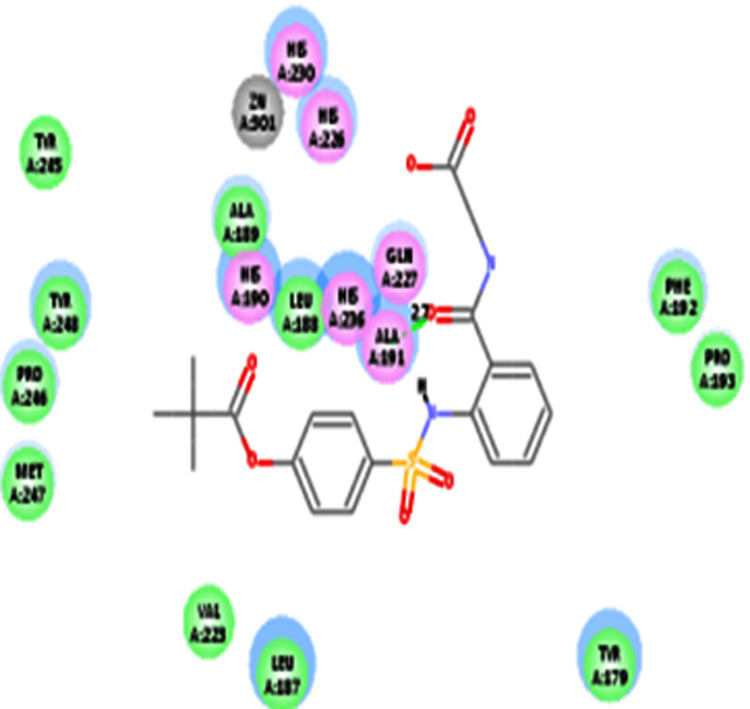
Interaction analysis of sivelestat (drug) with that of MMP-9 Hydrogen atoms have been excluded in the 2D figure to improve precision. Bond distances (BD) are indicated in angstroms (Å) unit. MMP-9: Matrix metalloproteinase-9

Similarly, ligand (sivelestat) showed the interaction energy (-58.7 kcal/mol) and also showed to interact with AA residue (Ala191) of MMP-9 (Table 6 and Figure 3).

**Table 6:** CDOCKER IE analysis of ligand (sivelestat) with MMP-9 using CDOCKER method IE: Interaction energy; AA: Amino acid; MMP-9: Matrix metalloproteinase-9

Ligand	CDOCKER IE (-kcal/mol)	Interaction of AA residue	Bond distance in Å
Sivelestat	58.7	Ala191	2.7

**Figure 4:**
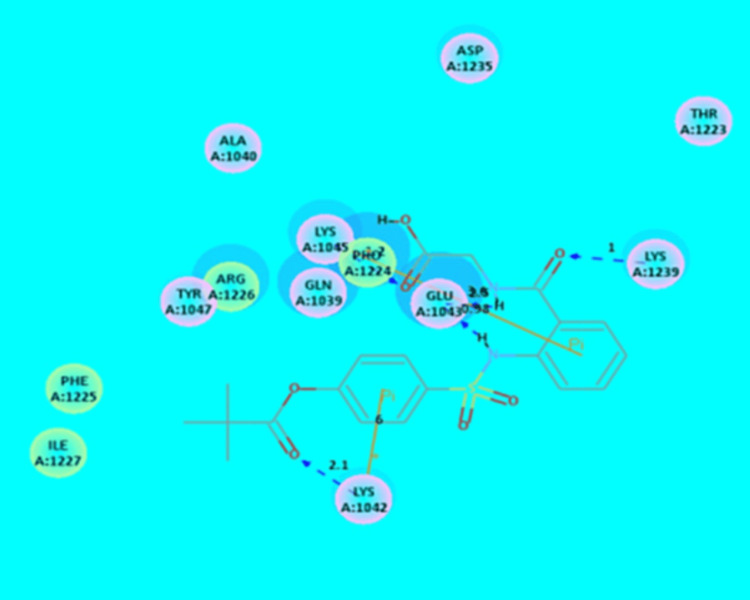
Interaction analysis of sivelestat (drug) with that of CvnsP2 protease Hydrogen atoms have been excluded in the 2D figure to improve precision. Bond distances (BD) are indicated in angstroms (Å) unit. CvnsP2: Chikungunya virus non-structural protein-2

In the present study, ligand (sivelestat) has shown the interaction energy (-47.8 kcal/mol) and also shown four AA residues (Lys1042, Glu1043, Lys1045 and Lys1239) interaction with chikungunya nsP2 protease (Table 7 and Figure 4).

**Table 7:** CDOCKER IE analysis of ligand (sivelestat) with CvnsP2 protease using CDOCKER method IE: Interaction energy; AA: Amino acid; ■: +-Pi interaction; CvnsP2: Chikungunya virus non-structural protein-2

Ligand	CDOCKER IE (-kcal/mol)	Interaction of AA residue	Bond distance in Å
Sivelestat	47.8	Lys1042	2.1
		Lys1042^■^	6.0
		Glu1043	0.98 and 2.5
		Lys1045	2.2
		Lys1045^■^	3.6
		Lys1239	1.0

**Figure 5:**
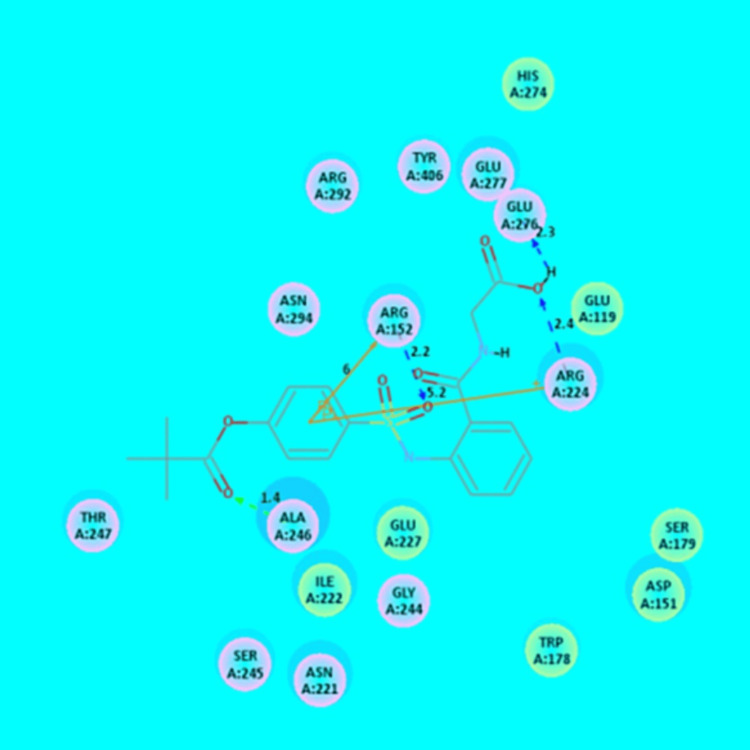
Interaction analysis of sivelestat (drug) with that of Influenza A virus neuraminidase Hydrogen atoms have been excluded in the 2D figure to improve precision. Bond distances (BD) are indicated in angstroms (Å) unit.

In the present study, ligand (sivelestat) has shown the interaction energy (-43.2 kcal/mol) and also shown four AA residues (Arg152, Arg224, Ala246 and Glu276) interaction with influenza neuraminidase (Table 8 and Figure 5).

**Table 8:** CDOCKER IE analysis of ligand (sivelestat) with Influenza A virus neuraminidase using CDOCKER method IE: Interaction energy; AA: Amino acid; ■: +-Pi interaction

Ligand	CDOCKER (IE) (-kcal/mol)	Interaction of AA residue	Bond distance in Å
Sivelestat	43.2	Arg152	2.2
		Arg152^■^	6.0
		Arg224	2.4
		Arg224^■^	5.2
		Ala246	1.4
		Glu276	2.3

## Discussion

**Figure 6:**
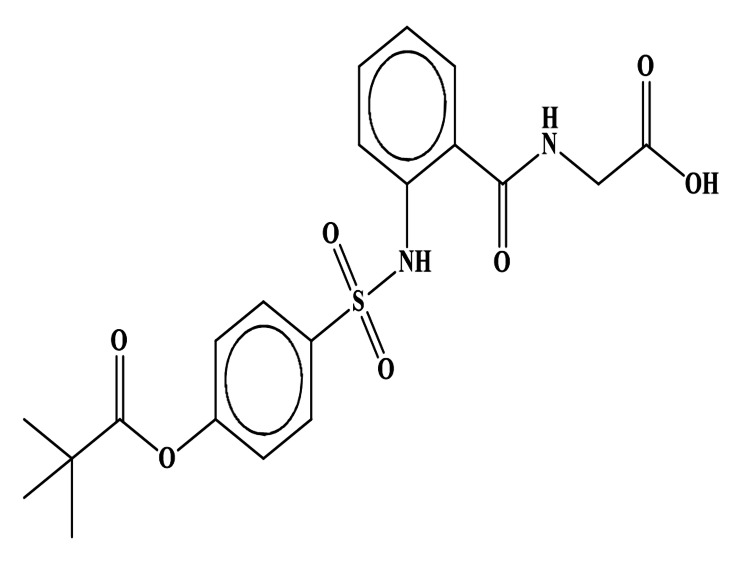
2D structure of sivelestat

Sivelestat is a competitive neutrophil elastase inhibitor in various species, including humans (Figure 6). It has advantages over the endogenous inhibitor alpha-1-proteinase, such as its low molecular weight and good anti-oxidant properties against oxygen free radicals [15].

Lipinski’s rule of five was utilized to know the molecular physicochemical properties of chosen ligands and violation of Lipinski’s rule of five is when LogA (Octanol-Water partition coefficient) value is more than 5; molecular weight is more than 500; Number of N, O (hydrogen bond acceptor) is more than 10; Number of OH and NH (hydrogen bond donor) is more than 5; and Number of the rotatable bond (Nrotb) is more than 15 [16]. Regarding bioactivity score, when the score is > 0 is active; between -5.0 to -0.0 is moderately active and greater than -5.0 is inactive [17]. ADME is an important screening tool to know oral bioavailability and is also commonly accepted in the drug discovery and development (DDD) programme, because of its unique characteristic nature [18,19]. STITCH analysis of the ligand (sivelestat) has shown interaction with the three Homo sapiens proteins (CELA 1, ELANE, and ICAM 1).

In the present study, ligand (sivelestat) had shown two AA residues (Ala167 and Pro221) interactions with MMP-2. The current result was in good correlation with the earlier report [20]. Matrix metalloproteases had been reported to possess both pro- and anti-inflammatory activity [2]. The ligand (sivelestat) had been shown to interact with the AA residue (Ala191) of MMP 9. The current result was in good correlation with the earlier report [20]. Similarly, sivelestat had been shown to inhibit MMP-9 mRNA expression levels in endometrial explants from the follicular phase. [21].

In the present study, ligand (sivelestat) has shown four AA residues (Lys1042, Glu1043, Lys1045, and Lys1239) interaction with chikungunya nsP2 protease. The current result was in good correlation with the previous report [7]. Similarly, ligand (sivelestat) has shown four AA residues (Arg152, Arg224, Ala246, and Glu276) interaction with influenza neuraminidase. The current result was in good correlation with the earlier report [7]. Moreover, inhibiting HNE is a new approach for treating inflammatory lung/respiratory diseases, such as H1N1 and SARS virus infections [10]. Furthermore, sivelestat had been reported to reduce the S1 signal of S-D614 protein and stop S-G614-driven entry into 293T-ACE2 in the presence of elastase at 100 μg/mL (concentration level) [22].

Limitations and future recommendations

The findings of the current study were based on molecular docking analysis, which provided new knowledge about the ligand (sivelestat) against MMP-2, MMP-9, CVnsP2 protease, and influenza neuraminidase enzyme inhibition activities. However, biochemical, cell-based assays and animal experiments studies are needed to confirm the ligand (sivelestat) as having potent inhibitory actions against MMP-2, MMP-9, chikungunya nsP2 protease, and H1N9 virus neuraminidase enzymes.

## Conclusions

In the current investigation, the ligand (sivelestat) had shown the potential to dock with all four targeted enzymes. However, the ligand (sivelestat) was predicated to exhibit a hepatotoxicity nature. Hence, the current investigation has highlighted new knowledge of sivelestat as potent MMP-2, MMP-9, chikungunya nsP2 protease, and influenza neuraminidase inhibition activities, which help in the management of inflammation and viral infection-associated diseases.
